# A Bibliometric Analysis of Research Trends in Biodegradation of Plastics

**DOI:** 10.3390/polym14132642

**Published:** 2022-06-29

**Authors:** Enoch Akinbiyi Akinpelu, Felix Nchu

**Affiliations:** Department of Horticultural Sciences, Faculty of Applied Sciences, Cape Peninsula University of Technology, P.O. Box 1906, Bellville 7535, South Africa; nchuf@cput.ac.za

**Keywords:** bibliometric, biodegradation, bibliographic coupling, co-citation, collaboration index, plastic, polymer, VOSviewer

## Abstract

The rapid growth in the production and application of plastic globally has resulted in plastic pollution with a negative impact on the environment, especially the marine ecosystem. One main disadvantage in the majority of polymers is disposal after a useful life span. Non-degradable polymers create severe difficulty in plastic waste management that might end up in landfills or wash into the ocean. The biodegradation of plastic waste is one solution to this critical problem of pollution. Hence, there is a need to consider the advancement of research in this subject area, in pursuit of a way out of plastic pollution. Thus, this study was designed to map the biodegradation of plastic-related research from 2000 to 2021. Statistical information on the topic was recovered from the Web of Science Core Collection and analysed using the bibliometrix package in RStudio statistical software, while data visualisation was conducted via VOSviewer. Our evaluation indicated that the amount of research on the biodegradation of plastic increased over the last decade, and the annual growth rate of publication trends was 11.84%. The study revealed that 1131 authors wrote the 290 analysed documents, with a collaboration index of 4.04. Cooper DG (*n* = 11) was the most relevant author, McGill University (*n* = 21) was the most active university, and the Journal of Polymers and the Environment (*n* = 19) the leading journal. The outcome of this study can guide prospective research and offer vital information for improving the management of plastic waste.

## 1. Introduction

Plastics are synthetic or semi-synthetic materials made through the polymerisation of organic and/or inorganic compounds [1,2]. Because of their vast domestic and industrial applications, there is a continuous increase in the production and consumption of plastics. As of 2017, 8.3 billion metric tonnes of original plastics have been produced, of which 76% became waste. Only 9% of plastic waste is recycled, 12% incinerated, and 79% ends up in landfills [3,4]. The majority of bio-based and petroleum-based plastics, such as polyethylene, polypropylene, polyvinyl chloride, and polystyrene, are not biodegradable. Consequently, the build-up of non-biodegradable plastics in soil resulted in decreased soil fertility, among other ecological and health challenges [5,6]. Similarly, plastic materials are difficult to manage and often end up in marine environments, beaches and surfaces of the shallow seabed, as well as the abysses. The degradation of large polymers leads to the proliferation of micro-plastic particles, which are poisonous to marine species. The micro-plastic particles aid the accumulation of other contaminants capable of impairing the feeding and growth of aquatic organisms, thus, damaging the robustness of marine species [3,7]. Therefore, researchers and other stakeholders have shown huge interest to surmount the build-up of non-biodegradable plastics in the environment [1,8].

The biodegradation of plastics depends on some factors, such as the composition or molecular weight of the plastics, environmental conditions (e.g., temperature, soil reaction), and the presence of suitable microorganisms [5,9,10]. Because most polymers are too large to pass through the microbial cell membrane, the biodegradation of polymers often focuses on increasing surface hydrophobicity to enhance microbial attachment [5,7]. Several microorganisms (such as bacteria and fungi) have been identified with the capacity to biodegrade both bio-based and petroleum-based plastics through the activities of extracellular and intracellular depolymerase [9]. These microorganisms produce exoenzymes that break down complex polymers into monomers small enough to pass through the semi-permeable cell membrane and then be used as carbon and/or energy sources under stress conditions [1,5]. However, due to the complexity of structure and insufficient knowledge about optimised conditions for rapid degradation of plastic polymers, the share of biodegradable plastics for commercial applications is very low [11]. Therefore, it is essential to evaluate the scope of research activities capable of providing information on existing works, trends, scientific collaborations, and their impacts.

The bibliometric technique is a useful tool for evaluating the trends and evolution of research in a specific field of interest [12,13]. It utilises different techniques to quantify the scientific impact of published papers over the years to provide ideas/concepts and directions for future work [14,15]. Bibliometric analysis is popular in the scientific community because it can be used for mapping foci and trends linked to authors, institutions, and countries, including the identification of research gaps in a particular niche [16,17]. The Web of Science (WoS) and Scopus are typically used as a source for the mining of bibliometric data [18,19]. Thus far, there is no bibliometric report on the biodegradation of plastics; therefore, we assessed published articles on the biodegradation of plastics and scientific mapping was developed to identify top authors, journals, institutions, and countries, as well as collaborative networks from 2000 to 2021. This study provides an abridged database of knowledge where researchers can access important information on trends and existing gaps for future research that could increase the commercial application of the biodegradation of plastics.

## 2. Materials and Methods

### 2.1. Data Mining

Numerous scientific databases can be mined for information on bibliometric analysis of research production in a specific field [18,20]. WoS is one of the most dependable and all-inclusive databases for bibliometric studies with millions of varying quality and high-impact scientific articles [16]. Thus, WoS core collection was mined for data on bibliometric analysis of research trends on biodegradation of plastics from 2000 to 2021. The search strategy was: TITLE ((“Biodegradation” OR “biological degradation” OR “microbial degradation” OR “enzymatic degradation”) AND (*plastic* OR “polymer”)). A set of documents (*n* = 456) were retrieved but the search was refined to scientific research articles (*n* = 374). Afterwards, manual validation to remove those articles that are not within the time range and do not relate with our focus by going through all the abstracts was performed, of which 84 articles were excluded. As such, 290 articles were found suitable for bibliometric analysis in this study. These documents were retrieved from WoS and saved for further processing.

### 2.2. Data Analysis

Rstudio (v.4.1.1) was used for data analysis. The mined data were uploaded into biblioshiny in Rstudio and analysed accordingly [21]. Consequently, VOSviewer software version 1.6.17 [22] was used to determine the institutions’ and authors’ collaboration networks on the biodegradation of plastic within the stipulated period.

## 3. Results and Discussion

### 3.1. Key Information about Data (2000 to 2021)

Details of the main information on the biodegradation-of-plastics-related research are shown in Table 1. The 290 articles exported from WoS were published in 145 sources by 1131 authors. The average years from the publication were 8.46, the mean citations/documents were 23.74, and mean citations/year/document was 3.105, with a total reference of 8620 for the 290 articles. Authors of multi-authored documents totalled 1119 and authors of single-authored documents were 12. Meanwhile, the number of single-authored documents was 13. The documents/author and authors/document are 0.256 and 3.9, respectively. Co-authors/documents were 4.57 and the collaboration index was 4.04. The value of the collaboration index signifies high research collaboration, which might be seen as signified by the average citations per document [23].

### 3.2. Annual Production Trends

**Figure 1:**
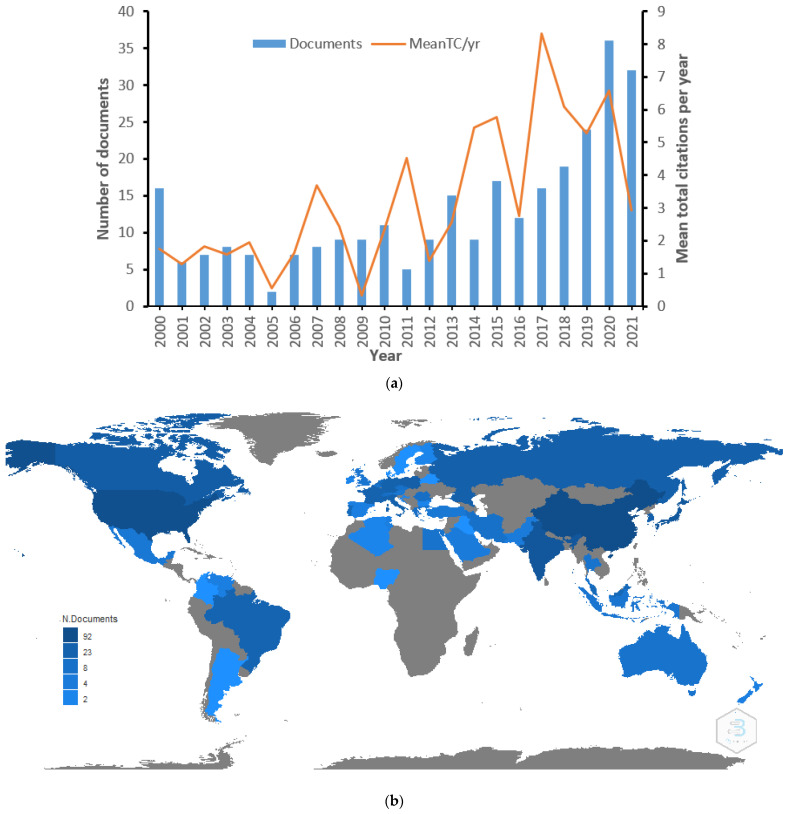
(**a**) Annual production and average citation per annum on biodegradation-of-plastics-related research from 2000 to 2021. (**b**) Country scientific production on biodegradation-of-plastics-related research.

Variations in the number of published research documents in a specific field are an important pointer for the developmental trend [24]. A plot of publications over time with statistical assessment would play a part in comprehending the research condition as well as prospective trends. About 55% of the documents were published between 2015 and 2021—Figure 1a. The highest publication was observed in 2020 with 36 documents, accounting for 12.4% of the total documents. The observed annual growth rate was 11.84%, an indication of a positive research trend in the biodegradation of plastics over time. The growing public attention to the circular economy of plastic pollution and the efficient biodegradation approach may be responsible for the upsurge in publications. Microorganisms with the potential for efficient plastic degradation could result in new prospects for palliating plastic pollution. Globally, the top five productive countries in biodegradation-of-plastics-related research are China with the highest number of documents (*n* = 92), followed by the USA (*n* = 82), India (*n* = 47), Japan (*n* = 45), and Canada (*n* = 31). Though China accounts for the largest production of plastic materials globally [25,26], China still makes a significant effort to remove plastic pollution through the implementation of policies and numerous funded projects. Countries with the greatest contribution are shown in Figure 1b, where the darker the blue colour, the greater the number of documents published. Most of the countries with the greatest contribution are economically developed or developing nations that ascribe huge significance to scientific research and they publish in journals indexed in WoS. Though the statistic from WoS may not be a true representation of all published scientific articles in a specific field, because some researchers are indifferent about the journals’ quality or they are interested in journals with fast publications only without considering visibility. Similarly, mean total citations per year were analysed over the same period and there is variation in the citation pattern. The highest citation was observed in 2017, followed by 2020, whereas the least citations were observed in 2005. Numerous factors can affect the citation of a research document. These include year of publication and open or paid access journal. Older publications are expected to have more citations than new ones [27]. However, papers in open access journals are easily available to other scholars and, thus, more cited than those in paid access. Citations are presumed to indicate the quality of the research, though in some cases, this is not so. The relationship between research quality and citations is a continuous debate in scientometrics [28].

### 3.3. Relevant Institutions and Authors

The most relevant institution in biodegradation-of-plastics-related research in terms of publication between 2000 and 2021 was McGill University, Canada, with 21 publications, followed by Universiti Putra Malaysia (*n* = 10), N.M. Emanuel Institute of Biochemical Physics, Russian Academy of Sciences (*n* = 7), Agricultural University of Athens (*n* = 6), and China Pharmaceutical University (*n* = 6). Details of the top 20 relevant institutions are shown in Table 2. Several studies have used bibliometrics to assess relevant institutions and authors in a specific field of interest [29,30].

The data from WoS revealed that 1131 authors wrote the 290 documents used for the analysis, with 3.9 authors per document—Table 1. The productivity and citation impact of authors were analysed as a function of h_index in Rstudio. The top five authors are Cooper DG (*n* = 11, total citations = 315, h_index =8), Nicell JA (*n* = 9, total citations = 236, h_index = 7), Briassoulis D (*n* = 6, total citations = 510, h_index = 6), Degli-Innocenti, F (*n* = 5, total citations = 191, h_index = 5), and Tosin, M (*n* = 5, total citations = 189, h_index = 5)—Table 3. Most of the Cooper DG publications are focused on the application of microorganisms, such as *Bacillus subtilis*, *Rhodococcus rhodochrous*, *Rhodotorula rubra*, and aerobic mesophilic microorganisms, consisting of bacteria, fungi, and yeast, for the degradation of different plastic materials [31,32,33,34]. H_index is a true indicator of researchers’ contribution and attainment but it is not suitable for assessing multidisciplinary fields [35,36]. Likewise, citations are a deficient method of quantifying an author’s impact in a field, since many issues affect the citation of a research publication [37]. In our analysis, although Cooper DG has the highest number of publications (*n* = 11), he is not among the top five researchers with the highest total citations. They are Wu WM (752 total citations), Yang Y (689 total citations), Yang J (644 total citations), Jiang L (641 total citations), and Briassoilis D (510 total citations). Year of publication is one of those factors influencing paper citations, notwithstanding the publication year (2002) of Cooper DG, Wu WM, Yang Y, Yang J, and Jiang L, whose publication year was 2014, as well as Briassoulis D with a publication year of 2007, attained higher citation than Cooper DG.

### 3.4. Relevant Journals

The distributions of research scope in a specific topic are best explained in journals and subject categories in bibliometric analysis [38]. A total of 125 journals published the 290 documents analysed. The top 20 productive journals represent 44% of the total publications. The most relevant journals on the subject are Journal of Polymers and the Environment (*n* = 19, IF = 3.667), Polymer Degradation and Stability (*n* = 15, IF = 4.63), Environmental Science and Technology (*n* = 10, IF = 9.028), Science of the Total Environment (*n* = 10, IF = 7.963), and International Biodeterioration and Biodegradation (*n* = 9, IF = 4.074), among others—Table 4. Environmental Science and Technology has the highest total citations (995), followed by Journal of Polymers and the Environment (total citations = 885), Science of the Total Environment (total citation = 440), Waste Management (total citations = 401), and Polymer Degradation and Stability (total citations = 396), among others. Notwithstanding, both Journal of Polymers and the Environment and Polymer Degradation and Stability have the highest h_index of 12 and publication year commencing from 2000.

The analysis of relevant journals showed that the studies in this field are more interdisciplinary, with a focus on the environment. Plastics are not only an environmental concern but also a hazard to microorganisms, soil, plants, human health, and the food chain, among others [39,40]. The increasing impact of plastics on the environment calls for an inclusive assessment of the ecological risk and effect on human health. Studies on the interaction among plastics, microorganisms, and human health may be a likely development in the biodegradation of plastics.

### 3.5. Bibliographic Coupling Analysis

**Figure 2:**
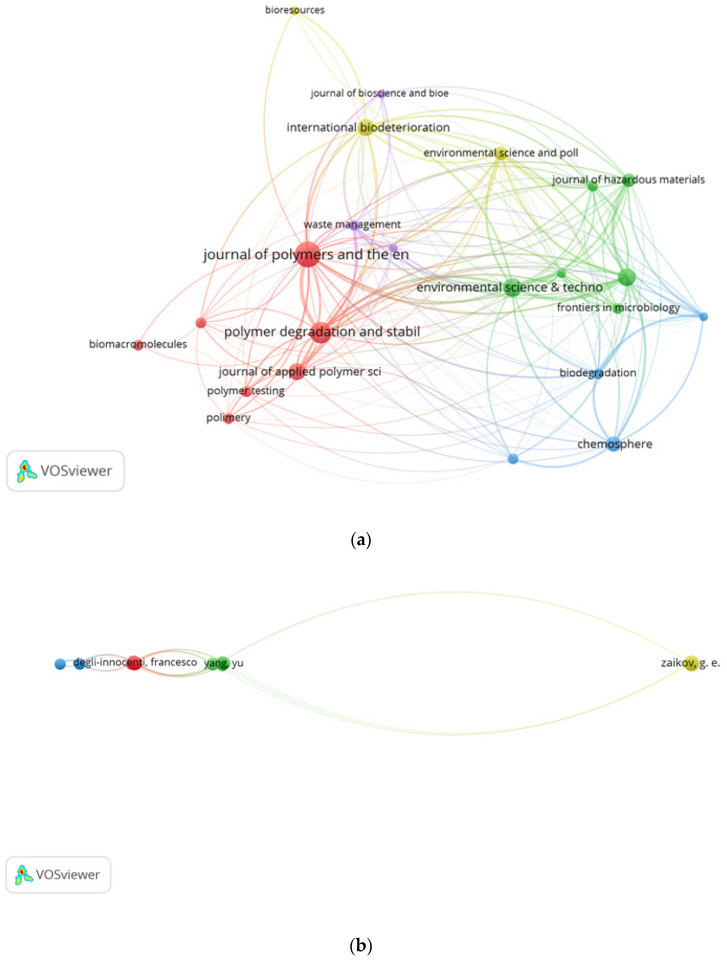

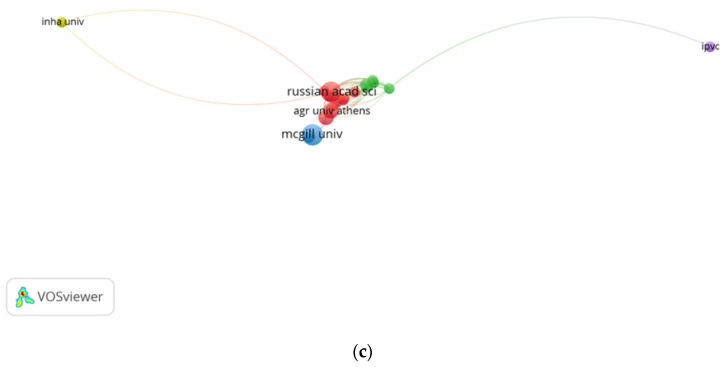
Bibliographic coupling analysis of (**a**) journals, (**b**) authors, and (**c**) institutions.

Bibliographic coupling analysis is a comparison measure that uses citation analysis to establish the relationship between documents based on the number of references shared [41]. This is to affirm the probability that the documents treat similar subjects and generate an information map of the research authors, institutions, and journals to show the collaborative network in biodegradation-of-plastics-related research. In this study, bibliographic coupling of authors, journals, and institutions was carried out using the full counting method in VOSviewer software. For bibliographic coupling analysis, we chose a minimum of three documents and 23 journals to meet the criteria. The bibliographic coupling link strength with other sources was calculated for each journal. The 23 journals were grouped into four clusters of 10, 6, 4, and 3 items in VOSviewer. Environmental Science and Technology has the highest total link strength of 574 with 11 documents and 995 citations, followed by the Science of Total Environment with 10 documents, 440 citations, and 564 total link strength—Figure 2a. Similarly, 22 authors meet the threshold. However, some of the 22 authors are not connected. The most connected authors consist of 17 authors. The 17 authors were classified into four clusters of 5, 5, 5, and 2 items. Cooper, D.G. has the highest total link strength of 1325 with nine documents and 211 citations, followed by Nicell, J.A. with 996 total link strength, seven documents and 132 citations—Figure 2b. For the bibliometric coupling analysis of institutions, the number of citations was five, and only 22 institutions meet the threshold. The 22 institutions were classified into five clusters of 11, 4, 3, 2, and 2 items. Stanford University has the highest total link strength of 385 with five documents and 767 citations, followed by Beihang University and Beijing Genomics Institute (BGI), Shenzhen with similar total link strength of 355 with three documents and 641 citations Figure 2c.

### 3.6. Collaboration Network Analysis

**Figure 3:**
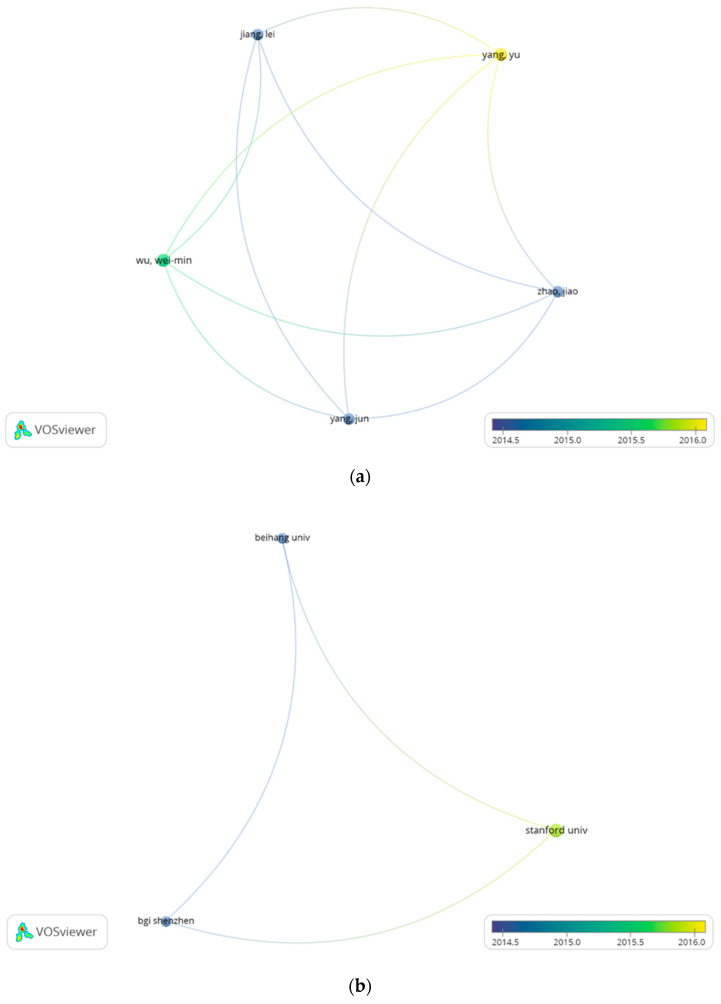

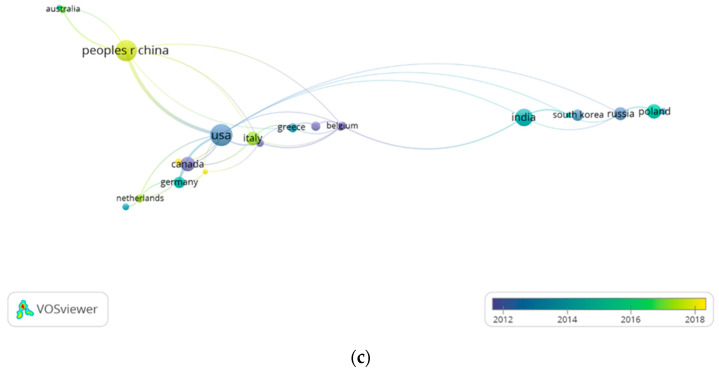
(**a**). Collaborative network analysis of authors in biodegradation-of-plastics-related research between 2000 and 2021. (**b**). Collaborative network analysis of institutions in biodegradation-of-plastics-related research between 2000 and 2021. (**c**). Collaborative network analysis of countries in biodegradation-of-plastics-related research between 2000 and 2021.

Collaboration network analysis can offer useful data for individual scholars, institutions, and countries seeking cooperation partners or groups to expand their field of research and realise the purpose of academic exchange [24]. This analysis reveals the reality of scientific research and academic communication among authors, institutions, and countries at various levels [42]. In this study, co-authorship of authors, institutions, and countries was analysed using the full counting method in VOSviewer. For the author’s analysis, the maximum number of authors per document was fixed as 25 and 1151 authors were observed. When the minimum number of documents per author was five and the minimum number of citations of an author was zero, only five authors met the threshold. The minimum number of documents per author was further reduced to three and the number of authors that meet this requirement increased to 22. For the 22 authors, the total strength of co-authorship links with other authors was calculated. Only 5 of the 22 authors in the network are well connected. They are Wu WM, Jiang L, Yang Y, Zhao J, and Yang J. Subsequently, the connection among the five authors was visualised—Figure 3a.

For the institution’s collaboration analysis, with a maximum of 25 institutions per document, 404 institutions were observed. Only five institutions met the threshold when the minimum number of documents per institute was five and the minimum number of citations was zero. Reducing the minimum number of documents per institute to three increased the number of institutions to 22. Most of the 22 institutions are not linked. The biggest group of connected institutions is three; they are Stanford University, Beihang University, and BGI Shenzhen and the connection is shown in Figure 3b. These analyses show that there is no strong collaboration among authors and institutions in the biodegradation-of-plastics-related research. More effort is needed to promote academic exchange in the biological degradation of plastic.

For the country’s co-authorship analysis, 52 countries were observed with the maximum number of countries per document being 25. With a minimum of five documents in a country and the minimum number of citations as zero, 18 countries met the selected criteria. A further reduction in the minimum number of documents of a country to three increased the number of countries that satisfy the requirement to 32. Some of the 32 countries are not connected. The biggest group of connected countries consists of 21 countries. The 21 countries were grouped into five clusters of six, four, four, four, and three items in VOSviewers. Clusters are grouped by the rate of shared co-occurrence terms representing each country. Terms with the same colour indicate they are strongly connected, thereby classified into the same cluster—Figure 3c. The more research publications a country has, the bigger the size of its circle; the larger the scale of the cooperation is, the thicker the connecting line [43]. The line connecting two items is the measurement of the level of cooperation between the two terms and is called link strength. The highest link strength between countries is 9 and found between China and the USA. The top three counties with the highest link strength are the USA (1906 citations, 24 link strength), China (1428 citations, 16 link strength), and Switzerland (202 citations, 7 link strength). This analysis shows there is no country from Africa among the collaborating countries; hence, much work is needed in this research area in Africa.

### 3.7. Co-Occurrence of Author Keywords

**Figure 4:**
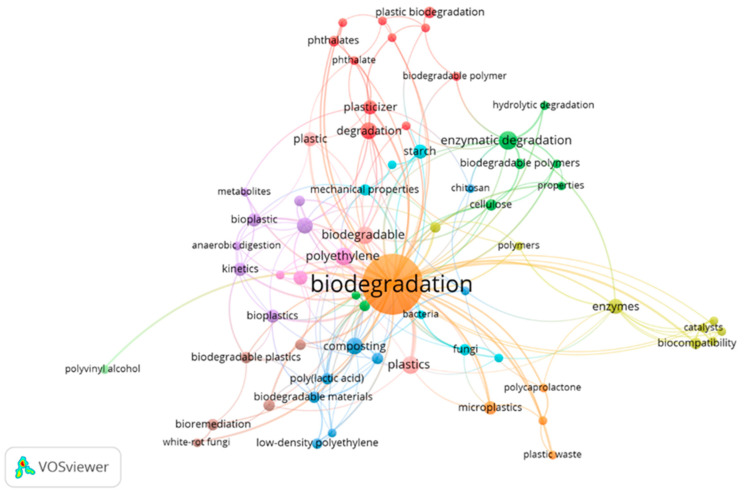
Co-occurrence of author keywords in biodegradation-of-plastics-related research between 2000 and 2021.

Co-occurrence analysis can be used to identify current topics and directions to observe and follow up the advances in scientific research and programs [44,45]. We analysed the co-occurrence of the author’s keywords via VOSviewer, shown in Figure 4. The size of a keyword indicates the number of publications in which it occurs, and the distance between two keywords gives a rough estimate of the relationship of the keywords. Using the full counting method, 779 keywords were observed. With the minimum number of occurrences of a keyword fixed at five, only 28 keywords meet the threshold. When we reduced the minimum number of occurrences of a keyword to three, 65 keywords met the requirement. The topmost keywords are: Biodegradation (125 occurrences, 106 link strength), Enzymatic degradation (12 occurrences, 6 link strength), Plastics (11 occurrences, 18 link strength), Polyethylene (11 occurrences, 17 link strength), Biodegradable (11 occurrences, 15 link strength), Composting (10 occurrences, 18 link strength), Degradation (10 occurrences, 6 link strength), Biodegradability (9 occurrences, 9 link strength), Enzymes (8 occurrences, 10 link strength), and Microorganisms (7 occurrences, 10 link strength). These keywords are a reflection of the research focus on the subject over two decades among scholars, and various researchers have utilised keywords to ascertain research directions in a specific field [17,18,20].

The 65 keywords were divided into eleven clusters of 11, 8, 8, 8, 7, 6, 5, 5, 3, 3, and 1 item in VOSviewer. Each cluster is represented with a unique colour to show the relatedness of the items in the cluster [46]. The keywords in identical clusters normally exhibit stronger links. The node size indicates the number of occurrences of a keyword and the attached lines show the existing connection. Cluster 1 centres on different plastic additives as well as their degradation and is identified by keywords, such as “2-ethylhexanol”, “phthalate”, “dehp”, “plasticizer”, “polyesters”, and “plastic biodegradation”, among others. Different methods of degradation of plastic materials are categorised in Cluster 2, which were identified by keywords, such as “enzymatic degradation”, “hydrolytic degradation”, and “biocomposite”, among others. Cluster 3 identifies plastic biodegradation material keywords, namely “biodegradable materials”, “biodegradation in soil”, “biofilm”, “composting”, and “chitosan”, among others. Microbial degradation of plastic is an environmentally benign method of managing plastic pollution [47]. Most research on the biodegradation of plastic is focused on the terrestrial environment, with several microorganisms, such as “bacteria”, “fungi”, and “microbes”, keywords identified in Cluster 6 [48,49,50]. There are limited reports of research on marine plastic sources degrading microorganisms [7,51]. Cluster 7 focused on various forms of plastic accumulation in the environment and their biodegradation. The keywords in this cluster include “microplastics”, “plastic waste”, “polystyrene”, “polycaprolactone”, and “biodegradation”. Previous studies have shown that microplastics are a carrier of toxic substances and other contaminants, which when consumed by organisms, such as fishes and seabirds, could affect their agility, feeding pattern, and reproductive system [52,53,54]. In addition, microplastics have been detected in foods, sea salts, and bottled water, including human stool samples and human placenta [55,56,57,58]. This suggests that microplastics can cause an imbalance in the intestinal microbiota, such as intestinal dysfunction and metabolic disorder [59,60]. Hence, studies on microbial activities on microplastics and their associated impact on human health are among the vital research topics needed.

### 3.8. Co-Citation Analyses

**Figure 5:**
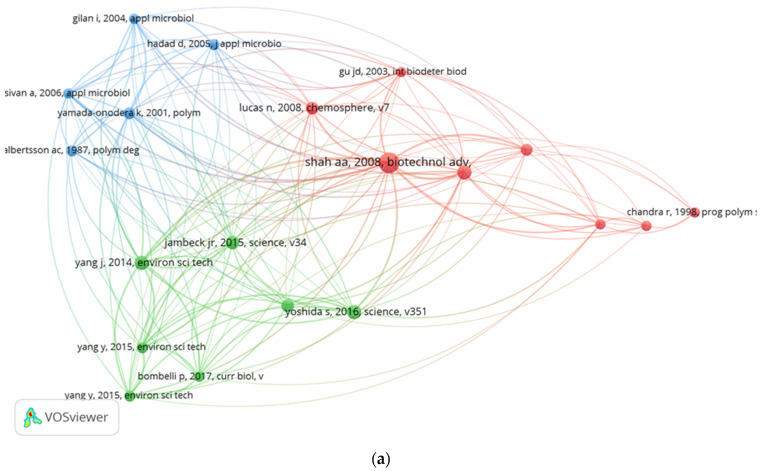

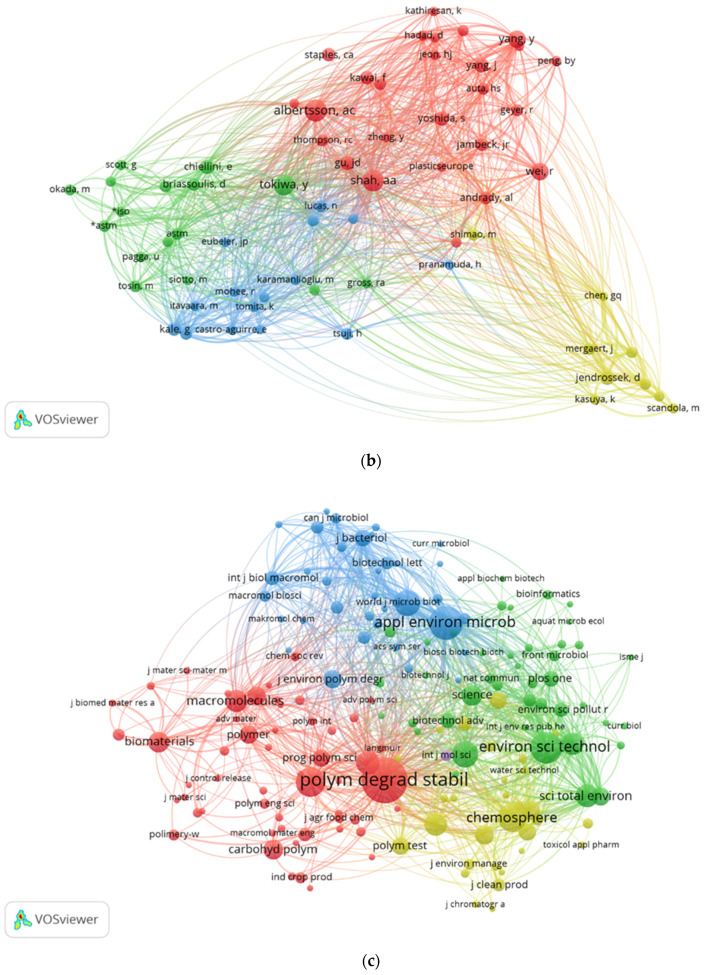
(**a**) Co-citation of cited references in biodegradation-of-plastics-related research between 2000 and 2021. (**b**) Co-citation of cited authors in biodegradation-of-plastics-related research between 2000 and 2021. (**c**) Co-citation of cited sources in biodegradation-of-plastics-related research between 2000 and 2021.

This is meant to determine the relationship of items based on how often they are cited together. Co-citation analysis provides important information on a topic in a specific field of research from the bulk of cited sources, authors, and references, that can assist in the evaluation of the most significant publications on a specific subject [24]. In this study, co-citations of cited references, cited sources, and cited authors were analysed—Figure 5. For cited references, the minimum number of citations of a cited reference was set as 10 and 23 cited references were found to meet the threshold out of 8659 cited references in the 290 publications analysed. Some of the 23 items are not linked. The biggest group of connected items consists of 21 cited references. The 21 items were grouped into four clusters of eight, seven, five, and one item in VOSviewer. The top five cited items are Shah, AA 2008 (40 citations, 109 total link strength), Tokiwa, Y 2009 (20 citations, 57 total link strength), Yang, J 2014 (19 citations, 85 total link strength), Yoshida, S 2016 (19 citations, 55 total link strength), and Jambeck, Jr 2015 (18 citations, 54 total link strength)—Figure 5a.

Similarly, for the co-citation of authors, 6716 authors were identified. With the minimum number of citations of an author as 10, 72 authors meet the criteria. Some of the 72 items are not connected. The biggest group of connected items consists of 66 authors, and they are classified into four clusters of 26, 16, 15, and 9 items. The top five are Shah, AA (52 citations, 437 total link strength), Albertsson, AC (58 citations, 365 total link strength), Tokiwa, Y (47 citations, 321 total link strength), Yang, Y (35 citations, 222 total link strength), and Wei, R (33 citations, 205 total strength)—Figure 5b.

In addition, cited sources returned 2923 sources. When the minimum number of citations of a source was 10, 165 sources met the requirement. The 165 items were classified into five clusters of 54, 43, 36, 31, and 1 item. The top five cited sources are Polymer Degradation and Stability (511 citations, 12064 total link strength), Applied Environmental Microbiology (287 citations, 8832 total link strength), Environmental Science and Technology (245 citations, 6361 total link strength), Journal of Applied Polymer Science (212 citations, 4120 total link strength), and Chemosphere (201 citations, and 5299 total link strength)—Figure 5c.

## 4. Limitations

This study presents the mapping of biodegradation-of-plastic-related research between 2000 and 2021. Nevertheless, the analysis might not be all inclusive of research publications on the topic, seeing as we centred on publications indexed in the Web of Science, excluding publications indexed in PubMed and Scopus, among other scientific databases. Similarly, it is likely that we did not use up all the possible keywords connected to the biodegradation of plastics within the specified time, and that might have created a bias in our analysis. Lastly, this study solely visualised the research trends on the topic but did not analyse the substance of every publication to determine the scientific quality or otherwise of the paper.

## 5. Conclusions

This study presented a comprehensive scientific mapping of global research on the biodegradation of plastic-related research from 2000 to 2021. About 290 publications were recovered from the Web of Science, written by 1131 authors. The Peoples’ Republic of China and the USA were the most prominent countries, with the highest publications and citations. In addition, the most relevant journals are the Journal of Polymers and the Environment and Polymer Degradation and Stability on the subject. We observed a low collaboration network among authors, with the strongest collaboration network between China and the USA, and no African country on the collaboration network analysis. This study suggests that more studies should be focused on the biodegradation-of-plastic-related research that elucidates the concurrent impact of microbial communities and microplastics on both the environment and human health. We believe this study would assist scholars interested in this field to identify potential collaborators, as well as provide valuable information for the effective planning and management of plastic contaminants, especially in the coastal and marine environment.

## Figures and Tables

**Table 1:** Major information on biodegradation of plastics-related research from 2000 to 2021.

Description	Results
Timespan	2000:2021
Sources (Journals, Books, etc.)	145
Documents	290
Average years from publication	8.46
Average citations per document	23.74
Average citations per year per doc	3.105
References	8620
DOCUMENT TYPES	
article	261
article; early access	5
article; proceedings paper	12
correction	4
editorial material	8
DOCUMENT CONTENTS	
Keywords Plus (ID)	811
Author’s Keywords (DE)	778
AUTHORS	
Authors	1131
Author Appearances	1325
Authors of single-authored documents	12
Authors of multi-authored documents	1119
AUTHORS COLLABORATION	
Single-authored documents	13
Documents per Author	0.256
Authors per Document	3.9
Co-Authors per Documents	4.57
Collaboration Index	4.04

**Table 2:** Top relevant institutions in biodegradation-of-plastics-related research between 2000 and 2021.

Institutions	Number of Articles
MCGILL UNIV	21
UNIV PUTRA MALAYSIA	10
NM EMANUEL INST BIOCHEM PHYS	7
AGR UNIV ATHENS	6
CHINA PHARMACEUT UNIV	6
UNIV MINHO	6
BEIHANG UNIV	5
KASETSART UNIV	5
MICHIGAN STATE UNIV	5
SHAANXI UNIV SCI AND TECHNOL	5
SHANGHAI JIAO TONG UNIV	5
STANFORD UNIV	5
UNIV FED SAO JOAO DEL REI	5
EINDHOVEN UNIV TECHNOL	4
INHA UNIV	4
JOHNS HOPKINS UNIV	4
LEHIGH UNIV	4
MENDEL UNIV BRNO	4
NATL CTR AGR UTILIZAT RES	4
NN SEMENOV CHEM PHYS INST	4

**Table 3:** Top relevant authors in biodegradation-of-plastics-related research between 2000 and 2021.

Authors	h_Index	g_Index	m_Index	TC	NP	PY_Start	AF
Cooper DG	8	11	0.381	315	11	2002	3.10
Nicell JA	7	9	0.333	236	9	2002	2.57
Briassoulis D	6	6	0.375	510	6	2007	2.50
Degli-innocenti F	5	5	0.556	191	5	2014	2.25
Tosin M	5	5	0.556	189	5	2014	1.50
Maric M	4	4	0.364	108	4	2012	0.90
Wu WM	4	4	0.444	752	4	2014	0.57
Yang Y	4	4	0.444	689	4	2014	0.78
Bellon-maurel V	3	3	0.13	118	3	2000	0.70
Erythropel HC	3	3	0.273	101	3	2012	0.70
Feuilloley P	3	3	0.13	118	3	2000	0.70
Gori R	3	3	0.375	69	3	2015	0.67
Jiang L	3	3	0.333	641	3	2014	0.45
Kasuya K	3	3	0.13	41	3	2000	0.67
Kim MN	3	3	0.143	62	3	2002	0.75
Kohler HPE	3	3	0.143	148	3	2002	0.56
Nalli S	3	3	0.143	146	3	2002	1.00
Silvestre F	3	3	0.13	118	3	2000	0.70
Watanabe T	3	3	0.214	25	3	2009	0.54
Yang J	3	4	0.333	644	4	2014	0.78

TC: total citation, NP: Number of publications, PY: Publication Year, AF: Article Fractionalised.

**Table 4:** Relevant journals in biodegradation-of-plastics-related research between 2000 and 2021.

Journals	h_Index	g_Index	m_Index	TC	NP	PY_Start
Journal of Polymers and the Environment	12	19	0.522	885	19	2000
Polymer Degradation and Stability	12	15	0.5217	396	15	2000
Environmental Science & Technology	10	10	0.4762	995	10	2002
Science of the Total Environment	8	10	0.4706	440	10	2006
International Biodeterioration & Biodegradation	9	9	0.4737	213	9	2004
Journal of Applied Polymer Science	7	9	0.3043	264	9	2000
Chemosphere	7	8	0.3043	209	8	2000
Environmental Science and Pollution Research	6	6	0.2609	185	6	2000
Journal of Hazardous Materials	5	5	0.3846	245	5	2010
Biodegradation	4	4	0.1905	108	4	2002
Biomacromolecules	4	4	0.1739	225	4	2000
International Journal of Biological Macromolecules	3	4	0.3000	89	4	2013
Polimery	3	4	0.1429	49	4	2002
Polymer Testing	4	4	0.1818	148	4	2001
Canadian Journal of Chemical Engineering	3	3	0.1304	23	3	2000
Environmental Technology	2	3	0.2500	16	3	2015
Journal of Bioscience and Bioengineering	3	3	0.1304	37	3	2000
Polymers	2	3	0.4000	33	3	2018
Waste Management	3	3	0.2500	401	3	2011
3 Biotech	2	2	0.2222	21	2	2014

TC: total citation, NP: Number of publications, PY: Publication Year.

## Data Availability

Not Applicable.
